# Supplementary material for: TRIM28-dependent SUMOylation protects the adult ovary from activation of the testicular pathway
Source: Nat Commun. 2022 Jul 29;13:4412. doi: 10.1038/s41467-022-32061-1 (PMC9338040; doi:10.1038/s41467-022-32061-1)
Supplement: Supplementary file 1 — Supplementary Information [file 41467_2022_32061_MOESM1_ESM.pdf]

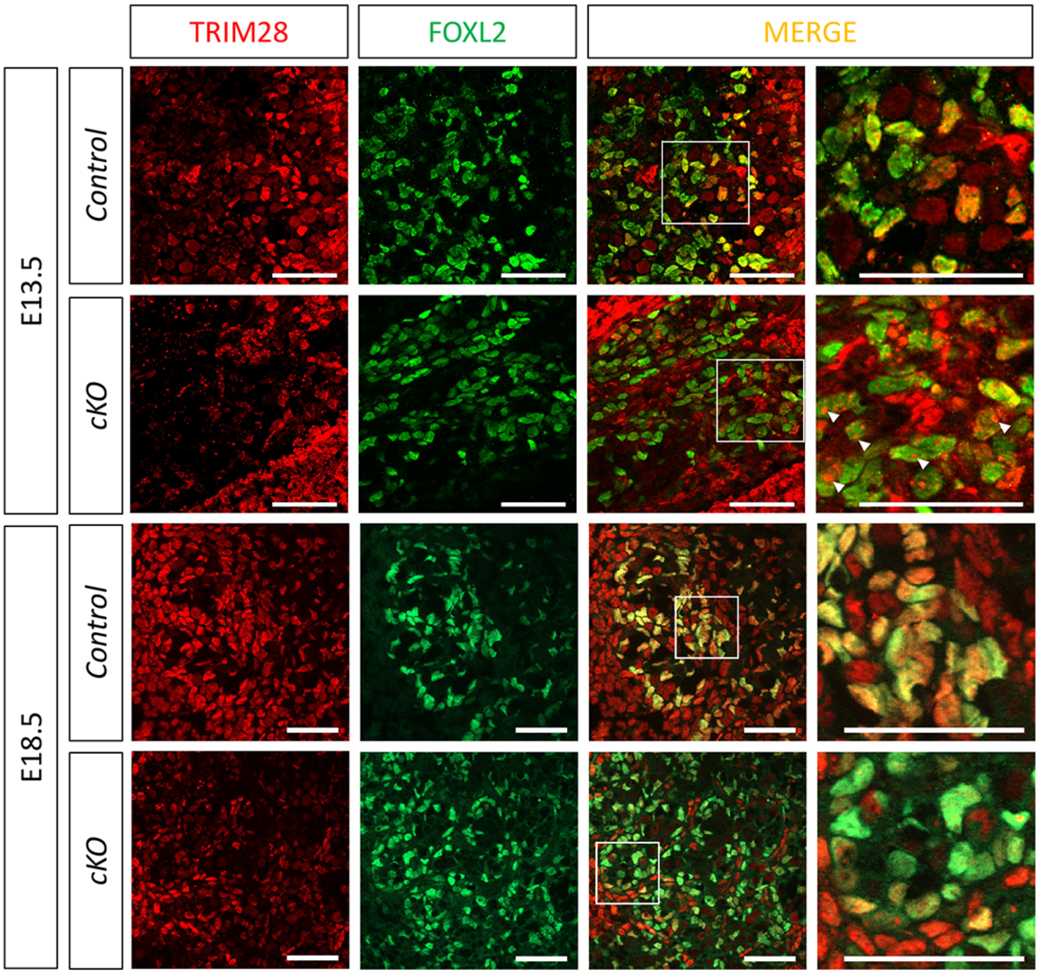

Supplementary Fig. 1. Expression of the TRIM28 protein in foetal pre-granulosa cells of control and mutant ovaries. At 13.5 dpc (E13.5) and 18.5 dpc (E18.5) in XX gonads, TRIM28 (red) is co-expressed with FOXL2 (green) in the nucleus of pre-granulosa cells. At E13.5, TRIM28 is in nucleoplasm and concentrated in nuclear dots that might be heterochromatin. At E18.5 TRIM28 is diffusely located within the nucleoplasm. In mutants, at E135, TRIM28 is in nuclear dots (white arrowheads) and appears decreased in the nucleoplasm of pre-granulosa cells. In E18.5 XX mutants, TRIM28 has nearly disappeared from the nucleus of pre-granulosa cells expressing FOXL2. Scale bar: 20µm. At least three independent biological replicates were analysed, and the images presented are representative of all replicates.

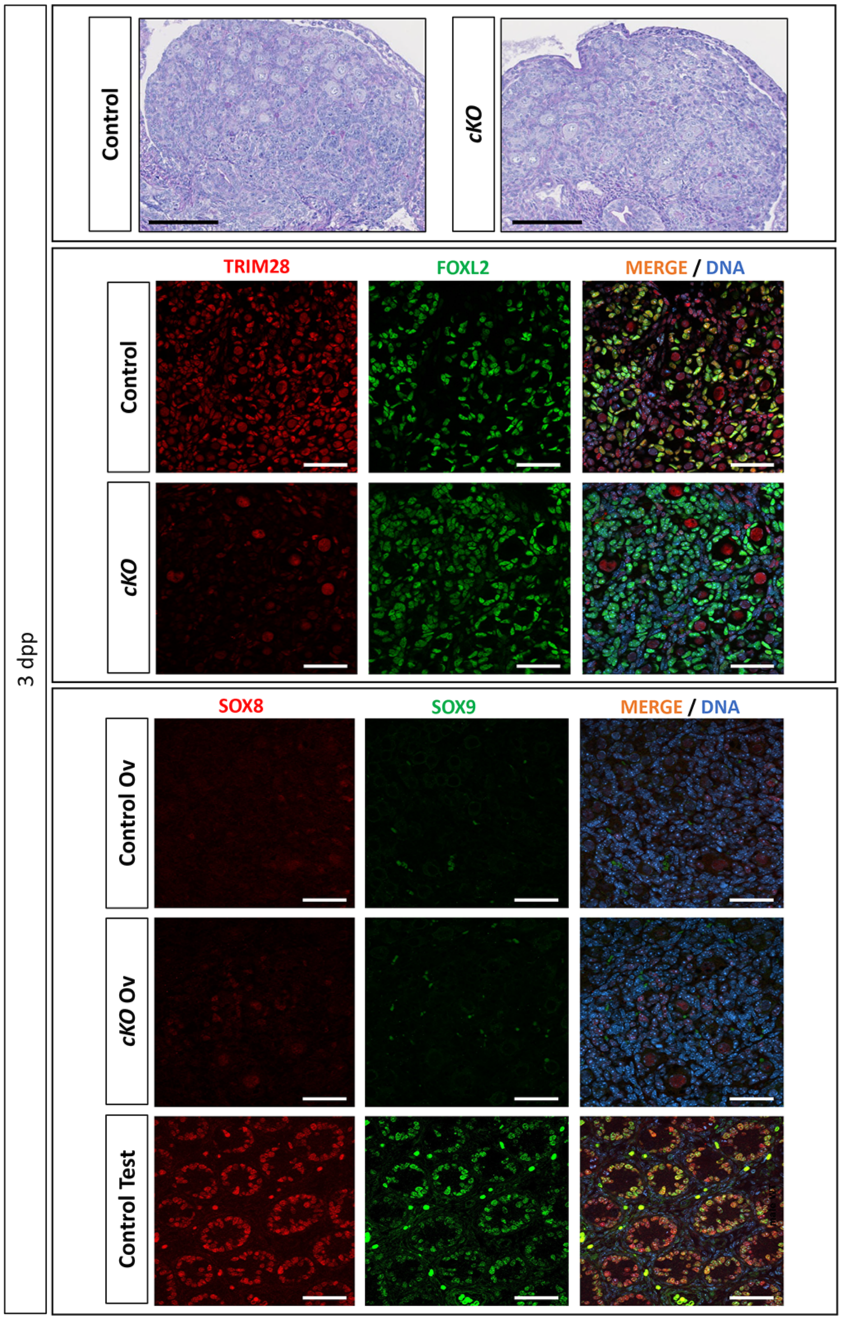

Supplementary Fig. 2. Upper panels: PAS staining showing that at 3 days post-partum (3 dpp), *Trim28*^{cKO} (*cKO*) ovaries display the same structures observed in control ovaries. Scale bar: 100µm. Lower panels: Double immunofluorescent staining shows that TRIM28 and FOXL2 are co-expressed in immature granulosa cells of 3 dpp control ovaries. In 3 dpp *Trim28*^{cKO} ovaries, TRIM28 signal has almost disappeared from cells that express FOXL2. Red staining in *Trim28*^{cKO} ovary sections corresponds to oocytes where *Trim28* is not deleted. Double staining for SOX8 and SOX9 shows that these Sertoli cell markers are not expressed in granulosa cells from 3 dpp control and *Trim28*^{cKO} ovaries (Ov). Conversely, in 3 dpp testes (Control Test), SOX8 and SOX9 are co-expressed in Sertoli cells that form seminiferous tubules. Interstitial staining corresponds to a secondary antibody artifact. Scale bar: 50µm. At least three independent biological replicates were analysed, and the images presented are representative of all replicates.

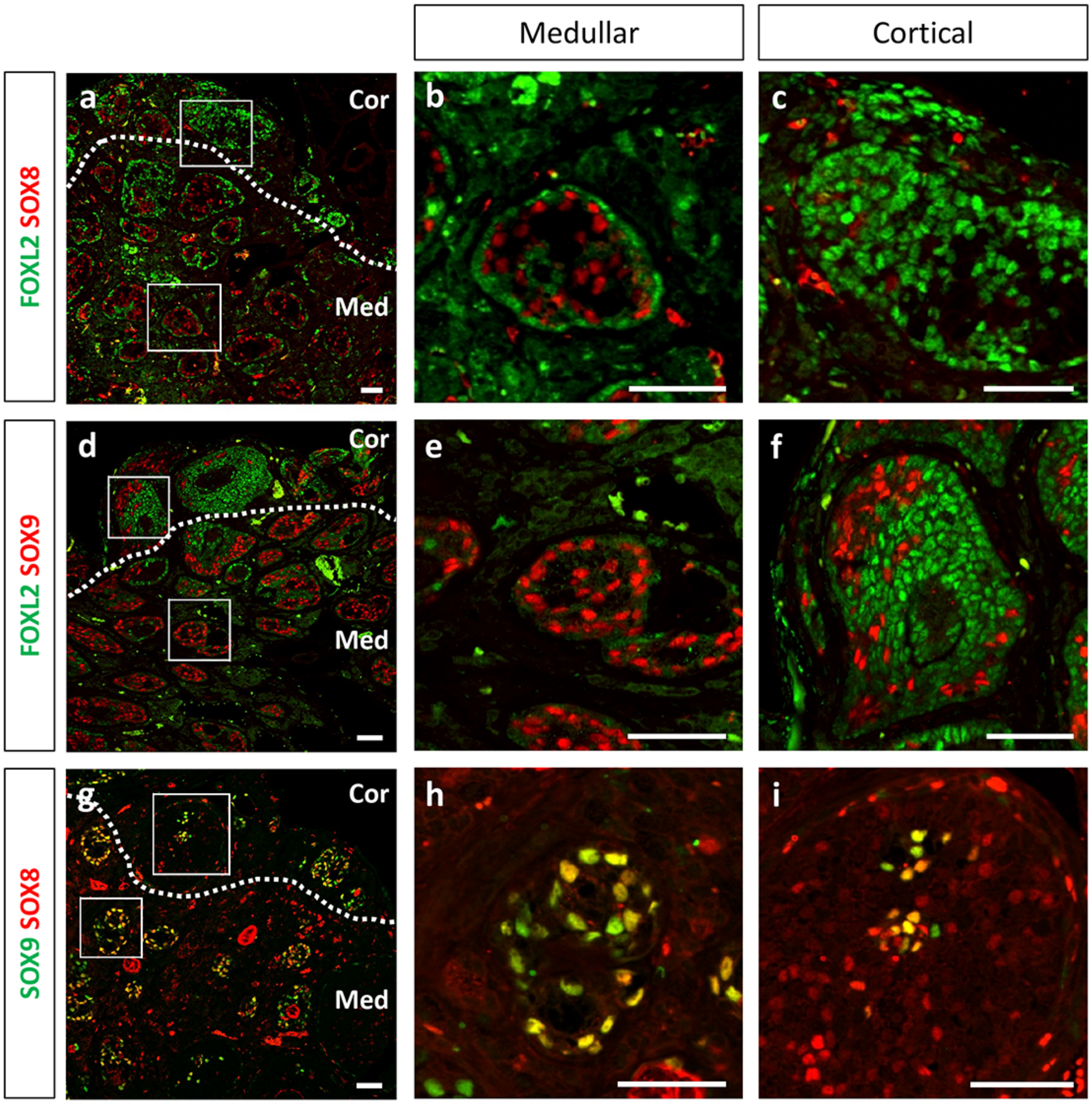

Supplementary Fig. 3. Double staining for FOXL2 and SOX8, FOXL2 and SOX9, and SOX8 and SOX9 in week 8 post-partum *Trim28*^{cKO} ovaries. Transdifferentiation of supporting cells is more advanced in the medullar (Med) than in the cortical area (Cor) (arbitrarily separated by a dotted white line). Boxed areas in the cortical and medullar areas are shown at higher magnification. Ovarian cortex still displays organized follicular structures that express FOXL2, although some isolated cells express SOX8 and/or SOX9. Conversely, in medullar areas, most follicular structures have disappeared and are reorganized in pseudo-tubules that express SOX8 and SOX9. SOX8 and SOX9 signals frequently overlap, but rarely with FOXL2. Strong interstitial staining is due to secondary antibody artifacts. Scale bar: 50µm. At least three independent biological replicates were analysed, and the images presented are representative of all replicates.

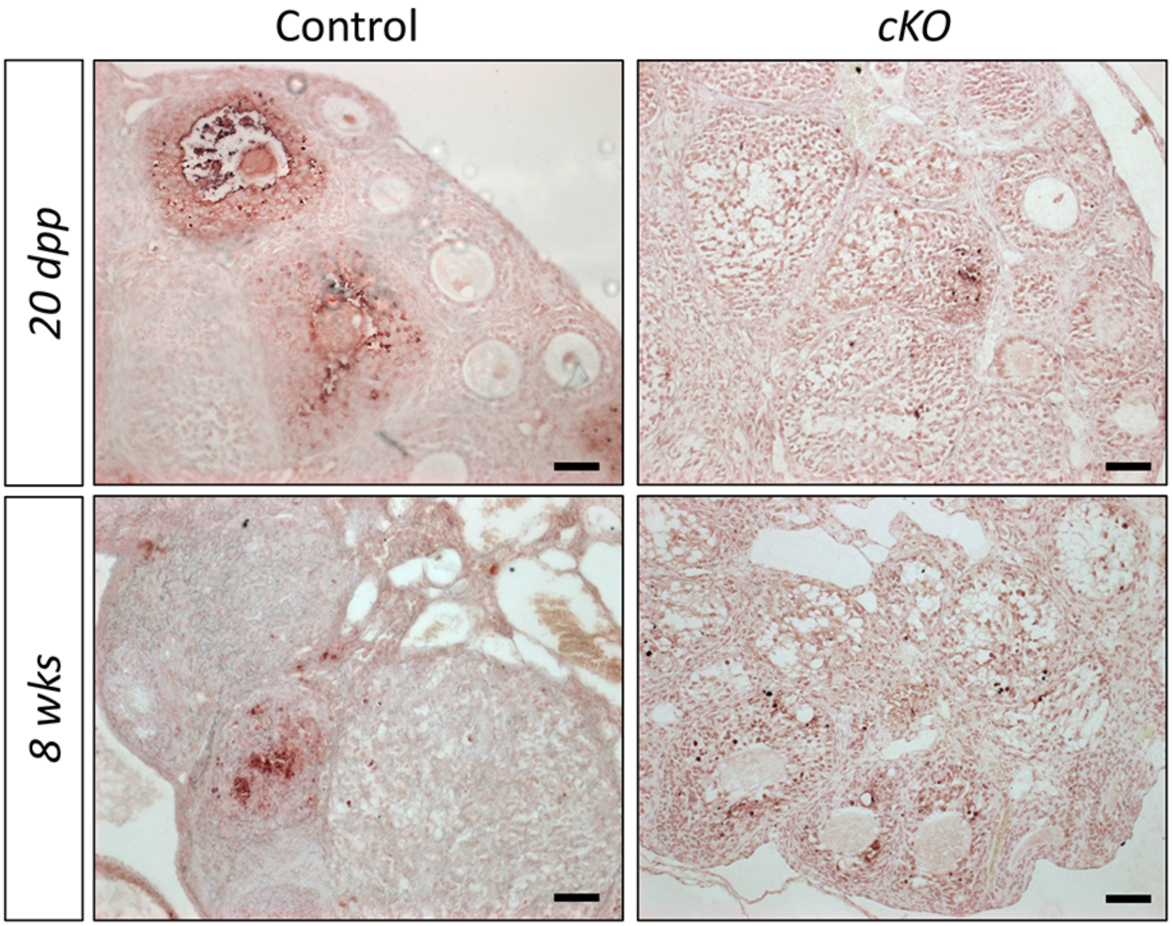

Supplementary Fig. 4. TUNEL staining of XX control and *Trim28*^{cKO} ovaries at 20 dpp and 8 weeks showing apoptotic nuclei (dark brown). No obvious difference was observed between follicles from control and mutant ovaries. Scale bar 50 µm. At least three independent biological replicates were analysed, and the images presented are representative of all replicates.

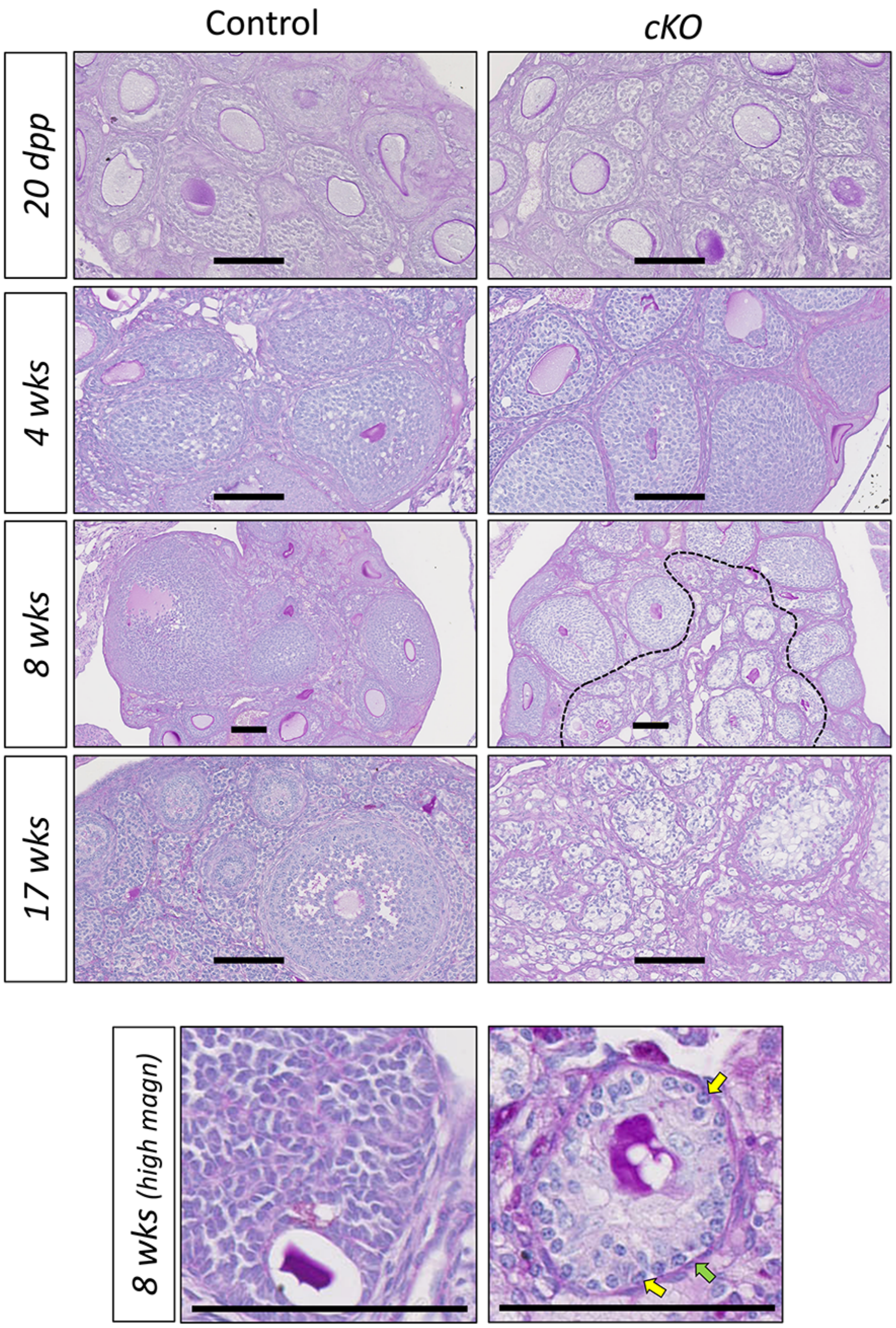

Supplementary Fig. 5. Progressive reorganization of ovarian follicles in *Trim28*^{cKO} (*cKO*) ovaries from 20 dpp to week 17 post-partum, visualized by PAS staining. In control, ovarian follicles develop normally, from pre-antral to pre-ovulatory follicles. At week 4 post-partum, ovarian organization is similar in control and *cKO* animals. At week 8 post-partum, multiple follicles reorganized in pseudo-tubules delineated by basal laminae are observed in the medullar region of *cKO* ovaries (arbitrarily delimited by a dotted black line). Cortical follicles contain apparently normal oocytes, while in medullar pseudo-tubules oocytes are degenerating or have disappeared. At week 17 post-partum, in *cKO* ovaries supporting cell transdifferentiation has spread to the entire ovary, and no oocyte can be detected. Bottom, High magnification of 8-week-old ovary tissue sections showing a preantral follicle and pseudo-tubules with features of Sertoli cells: nuclei with tripartite nucleoli (yellow arrows) and important deposition of basal laminae (green arrow). Scale bar: 100µm. At least three independent biological replicates were analysed, and the images presented are representative of all replicates.

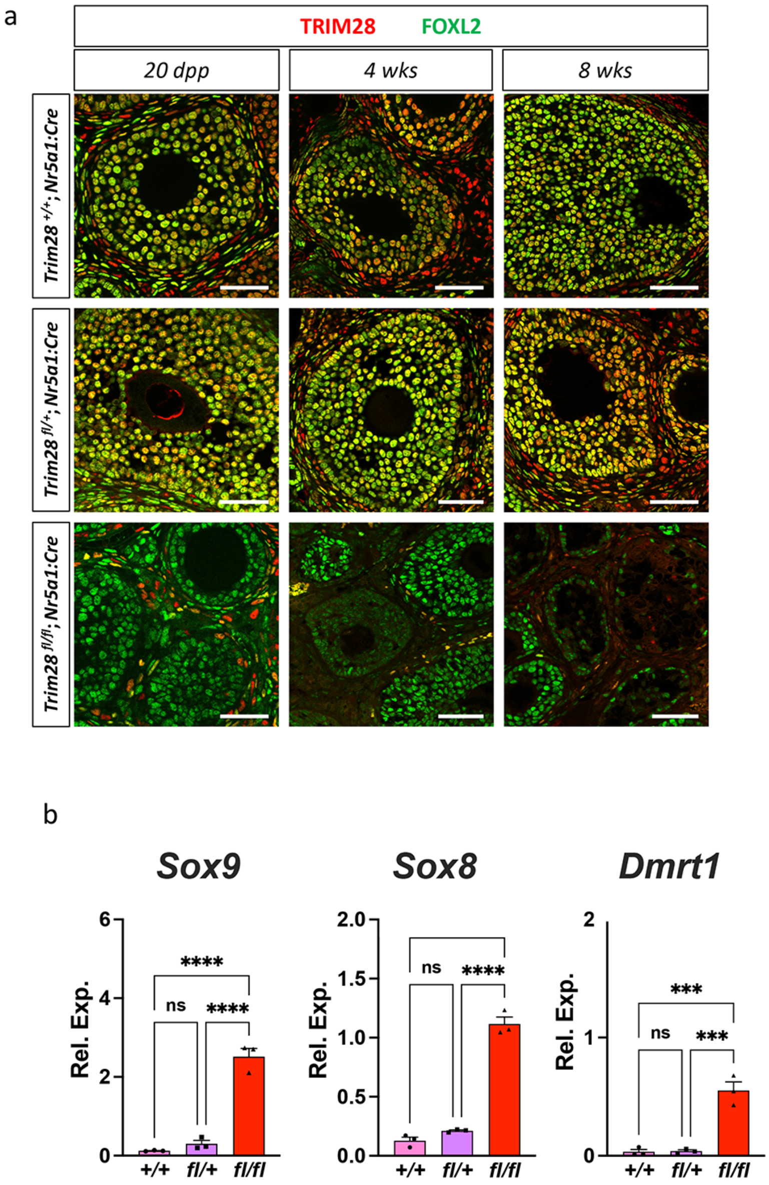

Supplementary Fig. 6. Effect of *Trim28* deletion heterozygosity on post-natal ovary. **a.** Double immunofluorescent staining for TRIM28 (red) and FOXL2 (green) in adult ovaries at 20 dpp, 4 weeks, and 8 weeks. No visible difference between wild type (Trim28$^{+/+}$; Nr5a1:Cre) and heterozygous mutant (Trim28$^{fl/+}$; Nr5a1:Cre) ovaries was observed in FOXL2 staining unlike in the homozygous mutant (Trim28$^{fl/fl}$; Nr5a1:Cre). Scale bar: 50µm. At least three independent biological replicates were analysed, and the images presented are representative of all replicates. **b.** RT-qPCR analysis of the Sertoli cell markers *Sox9*, *Sox8* and *Dmrt1* in 3-month-old ovaries from wild type (+/+: Trim28$^{+/+}$; Nr5a1: Cre), heterozygous mutant (fl/+: Trim28$^{fl/+}$; Nr5a1:Cre), and homozygous (fl/fl: Trim28$^{fl/fl}$; Nr5a1:Cre) mutant mice. Bars are the mean ±SEM, n=3 animals (gonad pairs). *P:* <0.0001 *(****)*, *0.0002(***)*, *0.0021(**)*, *0.032(*)* (one-way ANOVA with Tukey's multiple comparisons test). Source data are provided as a Source Data file. Details of the statistical analysis are provided in Source data file.

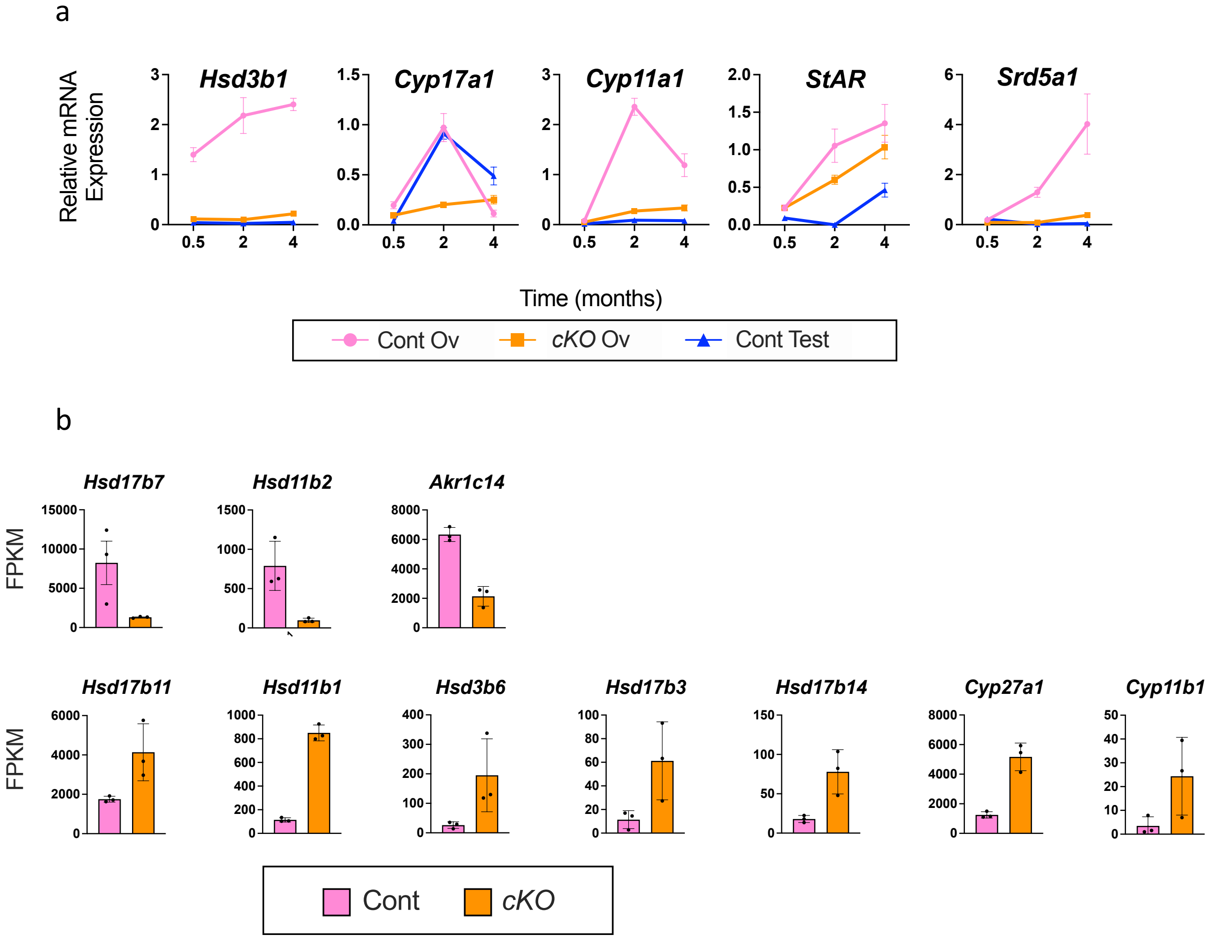

Supplementary Fig. 7. The expression profile of S6several genes involved in steroidogenesis is modified in *Trim28*^{cKO} ovaries. **a,** RT-qPCR analysis of key steroidogenesis genes from 0.5 to 4 months post-partum in control (Control Ov) and *Trim28*^{cKO} (cKO) ovaries, and control testes (Cont test). Bars are the mean ±SEM. For 0.5, 2 and 4 months: control ovaries n=5, 4, 4 animals (gonad pairs) respectively; cKO ovaries n=5 animals for each of the three conditions, control testes: n=3 animals for each of the three conditions. Source data are provided as a Source Data file. Details of the statistical analysis are provided in Source data file. Details of the statistical analysis are provided in Source data file. **b,** Expression of genes involved in steroidogenesis assessed in 7-month-old ovaries by RNA-seq (n=3 independent libraries). Values correspond to normalized read counts divided by the median of the transcript length in kb. Comparison between wild type and cKO samples was performed using the Wald test for differential expression proposed by Love et al.[1] Adj P Val: 1.36E-09 (Hsd17b7); 4.95E-20 (Hsd11b2), 2.69E-09 (Akr1c14); 6.72E-06 (Hsd17b11); 3.86E-55 (Hsd11b1); 6.35E-08 (Hsd3b6); 0.002023972 (Hsd17b3); 0.002226639 (Hsd17b14); 2.11E-22 (Cyp27a1); 0.004162312 (Cyp11b1). │Log2FC│>1. Values are the mean ± SD. Data and statistics are available in Data S1

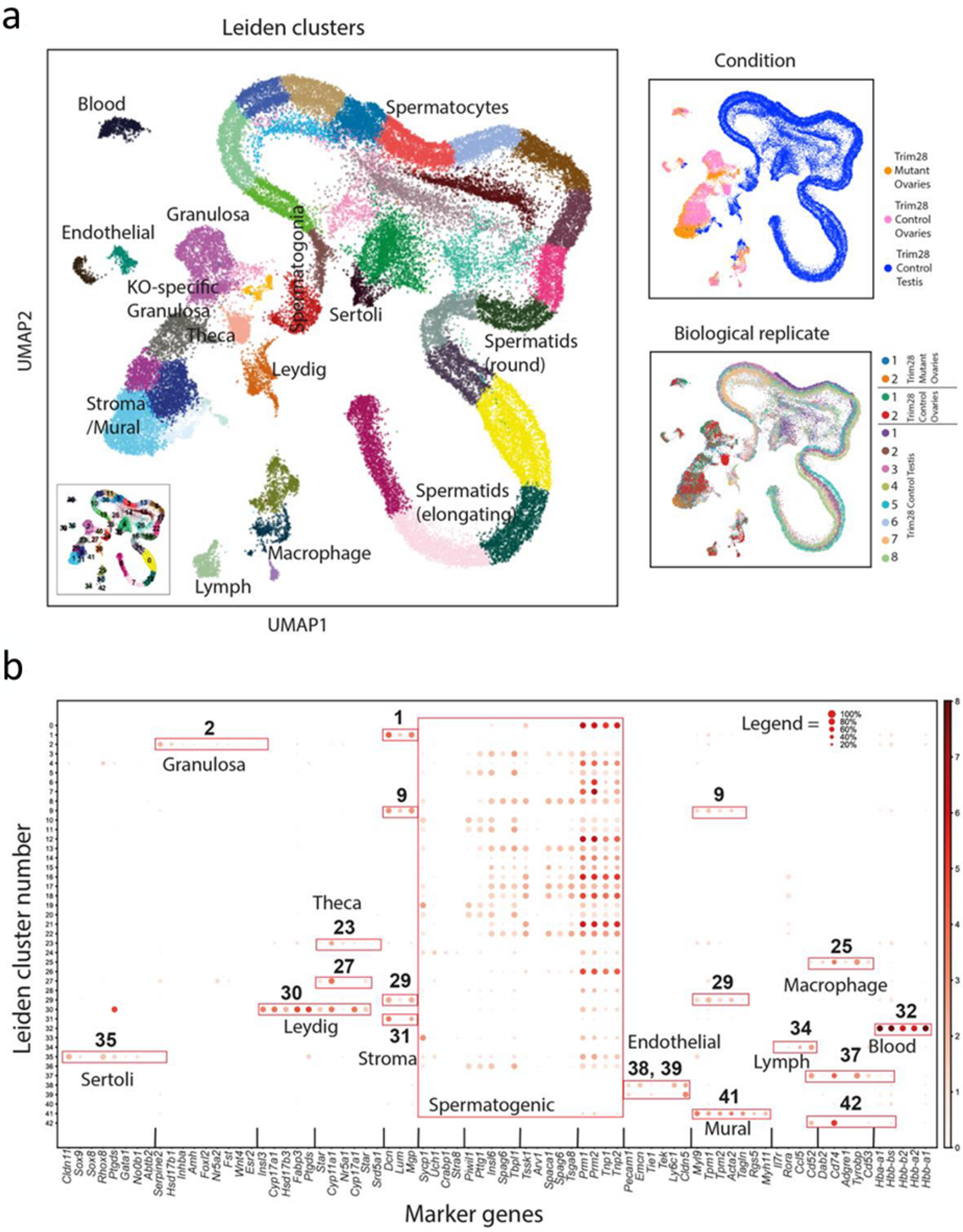

Supplementary Fig. 8. Single-cell atlas of 8-week-old *Trim28*^{cKO} and control ovaries and testes. **a,** UMAP representations of single cells in the transcriptional space coloured according to the Leiden clustering (left), condition (i.e. ovary/testis and mutant/control; upper right) and biological replicate (lower right). **b,** Expression of marker genes in the different Leiden clusters (1 to 42). Details in Data S3, tab df_gene_clusters). The colour of each dot indicates the mean expression within that cluster based on the normalized plus log transformed counts. The dot size represents the fraction of cells expressing that gene. Note that not all markers are specific, for example *Star* is a steroidogenic marker of both Leydig and theca cells. Cells from clusters 2 and 35 are granulosa/mutant and Sertoli cells, respectively, and were used in the subsequent analyses.

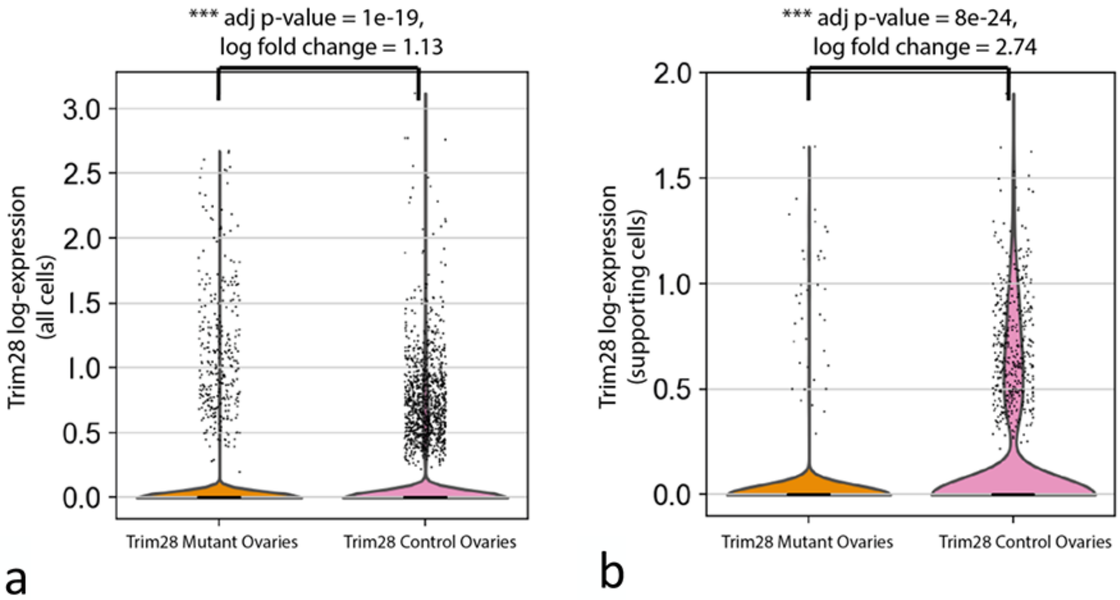

Supplementary Fig. 8. *Trim28* expression is decreased in the cKO mutant compared with control ovaries. Violin plots show the expression of *Trim28* in all ovarian cell types (**a**) and only in supporting cells (**b**). The Wilcoxon rank sum test (one-sided) across all genes to calculate the multiple-testing adjusted p-values and log fold-changes demonstrated that *Trim28* expression levels are lower in mutant than control ovaries.

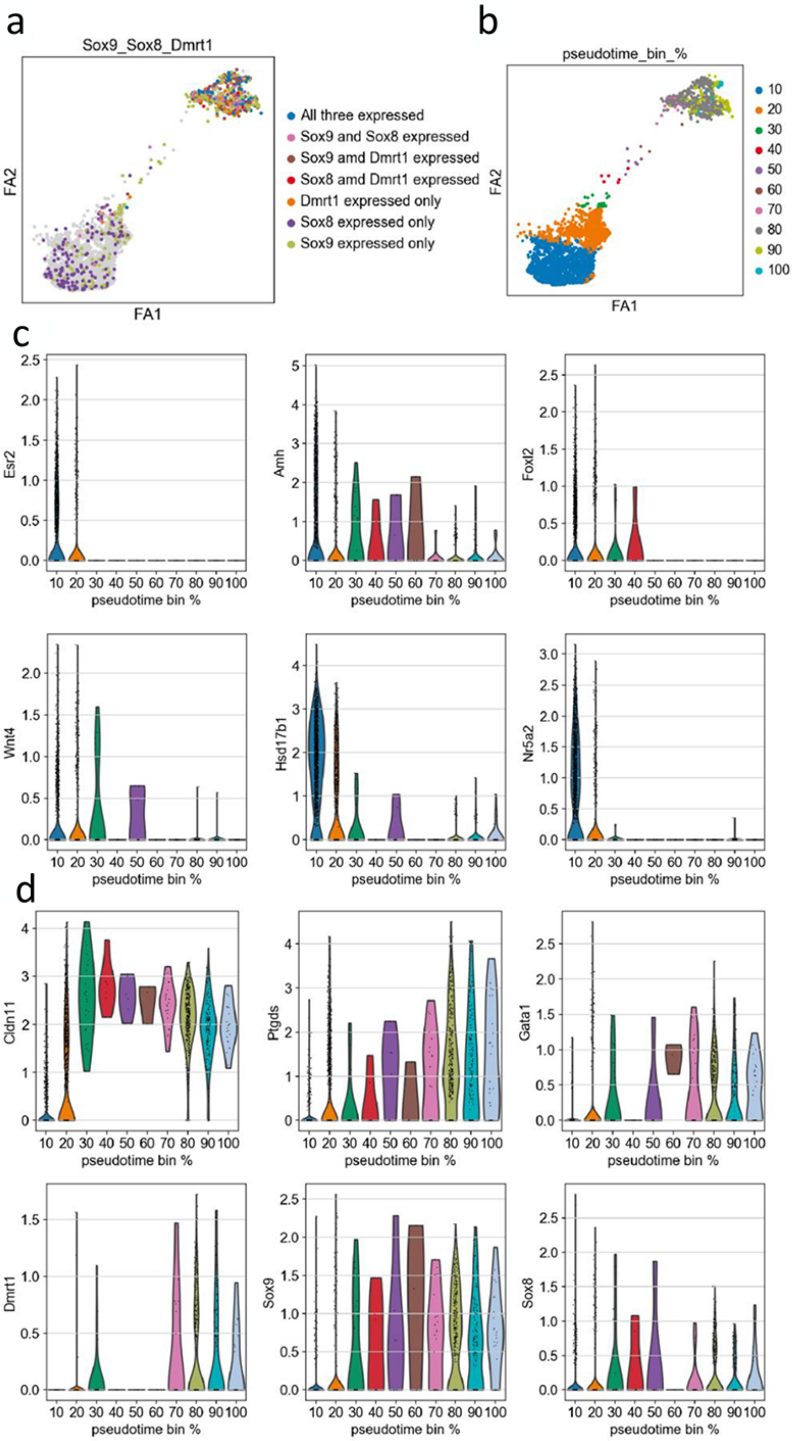

Supplementary Fig. 10. Expression of granulosa and Sertoli marker genes in supporting cells (i.e. granulosa, intermediate, and Sertoli) across the binned pseudo-time.

a, Representation of the binary (yes/no) expression of *Sox9*, *Sox8* and *Dmrt1* on the force directed graphs (FDG) from **Figure 1**. **b,**. Ten different pseudo-time bins are displayed on the FDG representing the pseudo-time value as a percentage, p, as follows: $0 \le p \le 10$, $10 < p \le 20$, $20 < p \le 30$, $30 < p \le 40$, $40 < p \le 50$, $50 < p \le 60$, $60 < p \le 70$, $70 < p \le 80$. $80 < p \le 90$, $90 < p \le 100$. Violin plots showing the expression of selected granulosa (**c**) and Sertoli (**d**) cell markers across the binned pseudo-time.

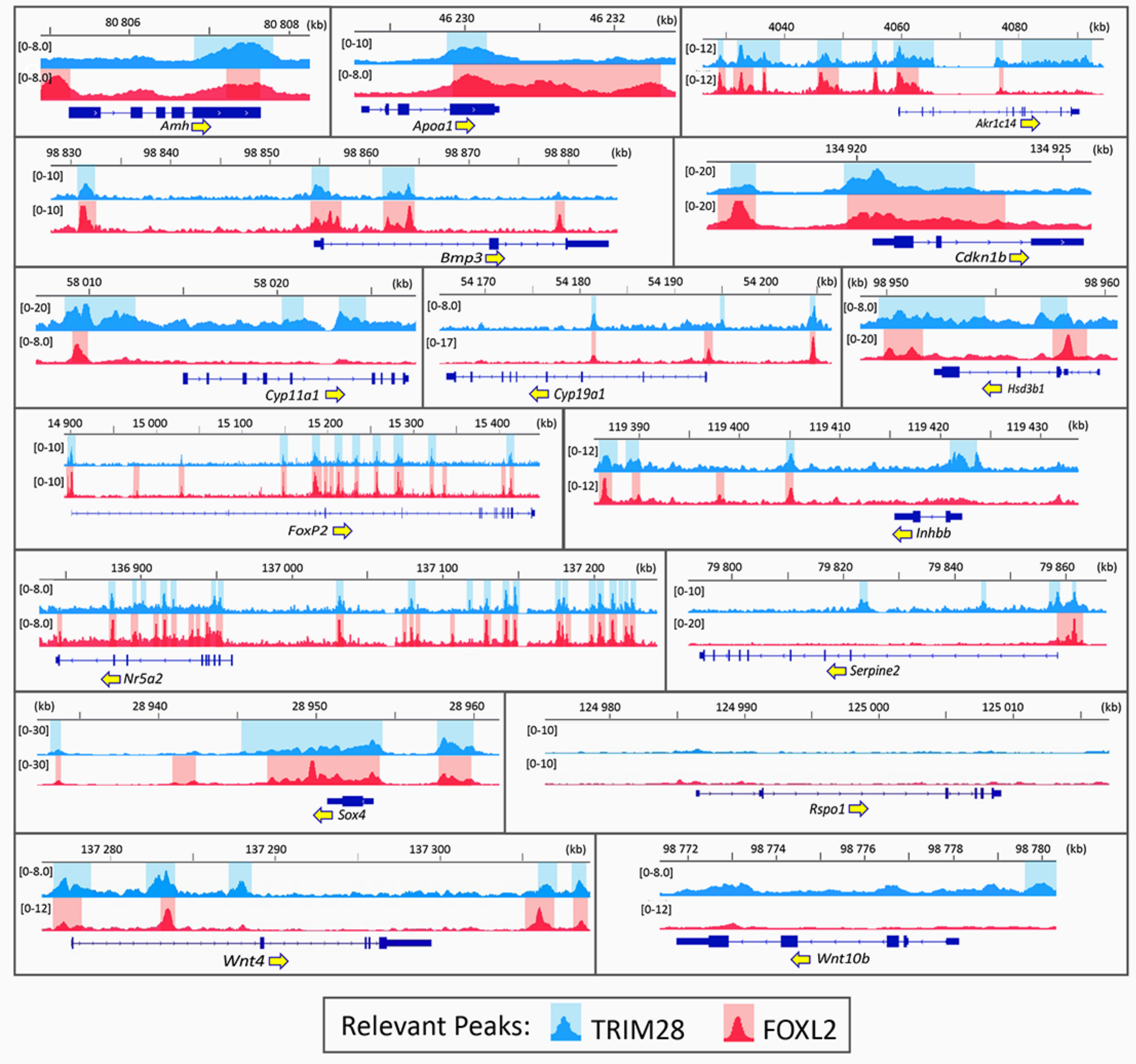

Supplementary Fig. 11. Distribution of TRIM28 and FOXL2 ChIP-seq peaks on genes expressed in granulosa cells that are downregulated in *Trim28*^{cKO} ovaries. Relevant ChIP-seq peaks are highlighted by a blue (TRIM28) or red (FOXL2) background.

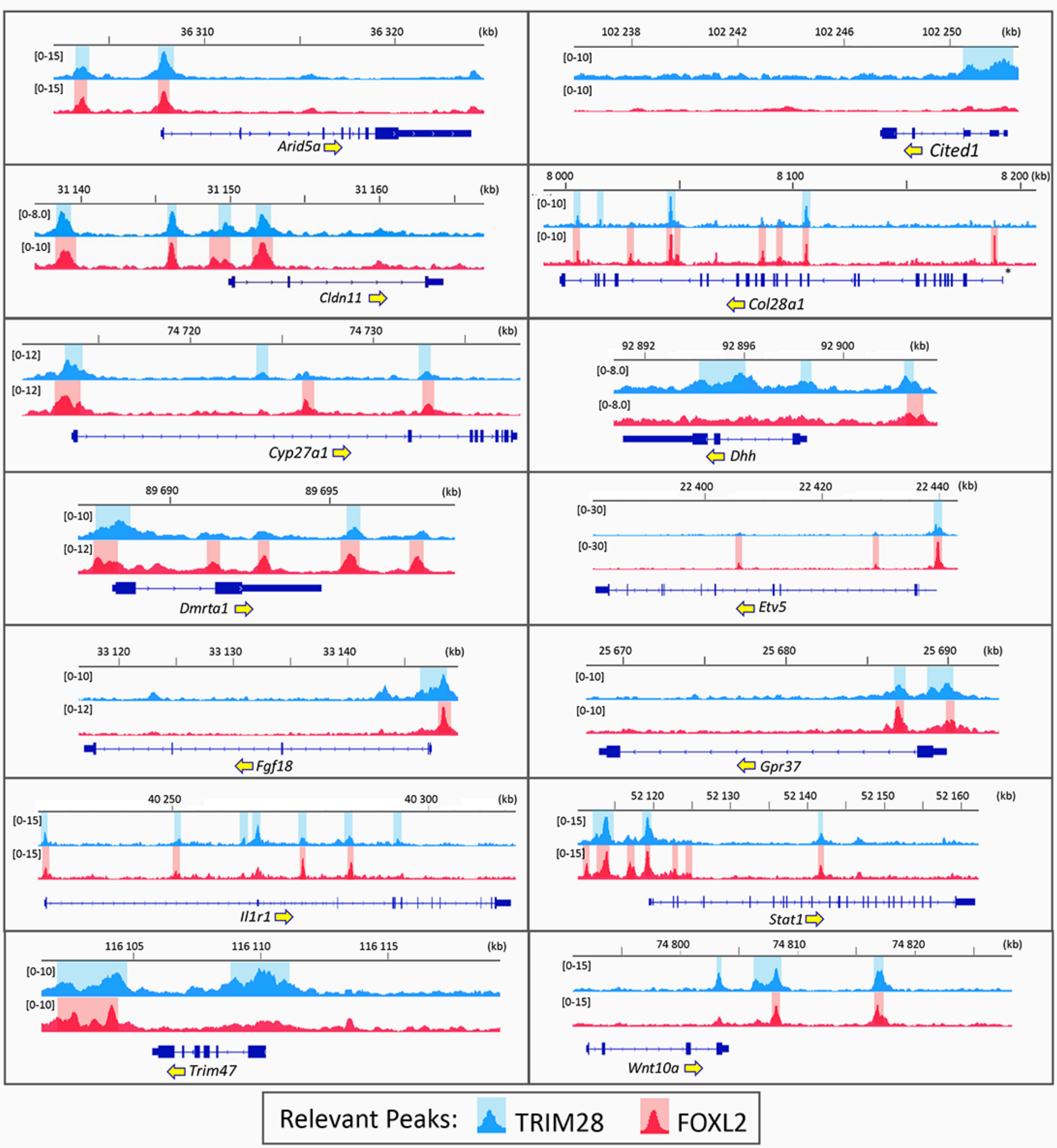

Supplementary Fig. 12. Same as in fig. S11 but for genes expressed in Sertoli cells that are upregulated in *Trim28*^{cKO} ovaries. Relevant ChIP-seq peaks are highlighted by a blue (TRIM28) or red (FOXL2) background.

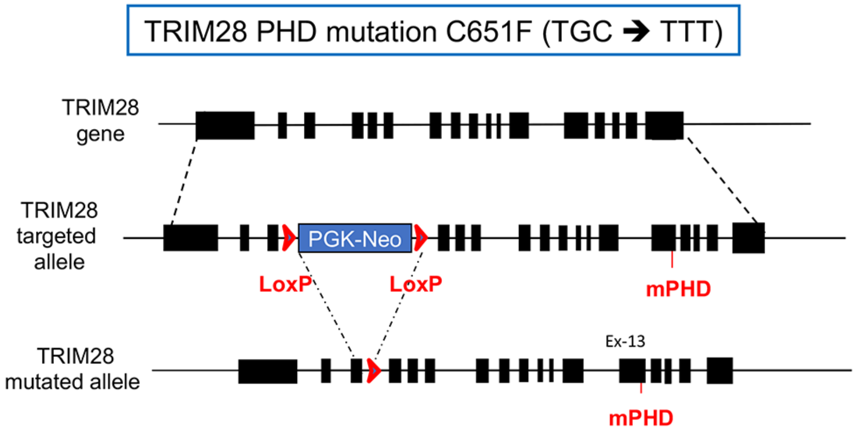

Supplementary Fig. 13. Targeted mutation (C651F) of the PHD domain of TRIM28. Diagram showing the genomic map of the mouse *Trim28* gene; the targeting constructs to introduce the C651F mutation in the PHD domain (exon 13); Cre-mediated excision of the loxP-site-flanked sequences. Exons are represented as black boxes and introns as connected lines. The loxP sites are represented by red triangles and the PGK-neo cassette is indicated.

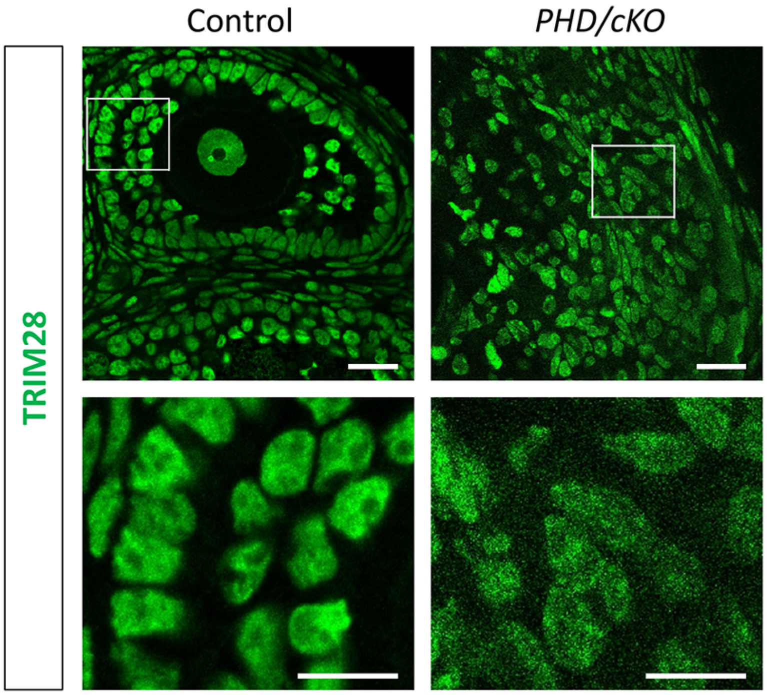

Supplementary Fig. 14. Immunofluorescent staining for TRIM28 in *Trim28*$^{+/+}$ control and *Trim28*$^{Phd/cKO}$ ovaries showing that the mutant TRIM28^{C651F} protein is effectively produced and localizes in the nucleus. Boxed areas are shown at higher magnification (lower panels) Compared with control, high magnification view of TRIM28^{C651F} *(PHD/cKO)* shows a lower nuclear staining because only the mutated allele is expressed. Scale bar: 10µm. At least three independent biological replicates were analysed, and the images presented are representative of all replicates.

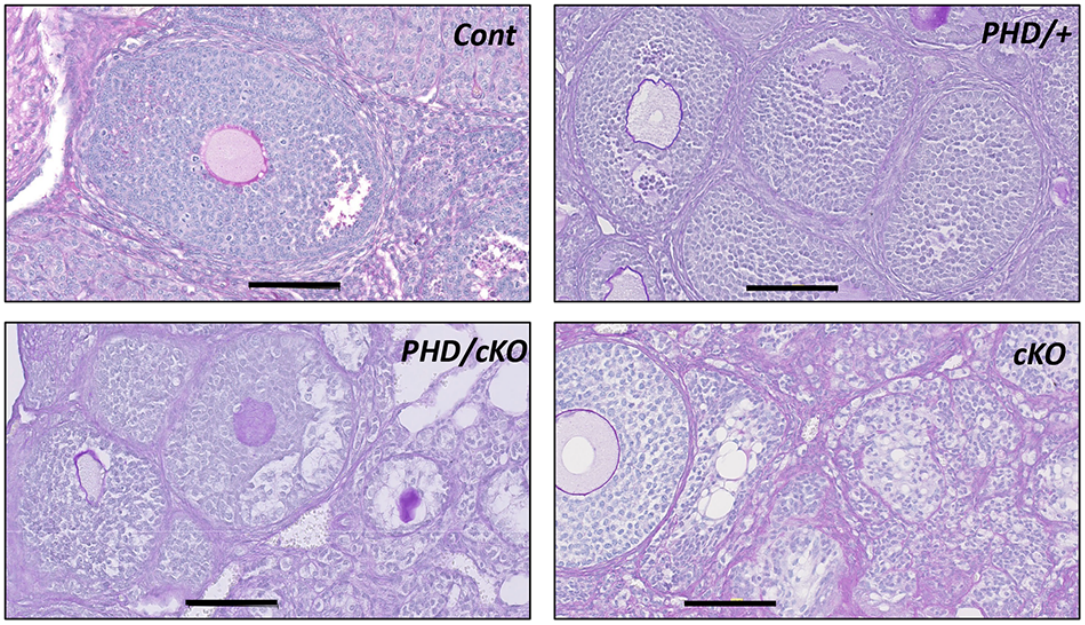

Supplementary Fig. 15. PAS staining of medullar regions of 8-week-old ovaries showing normal follicular structures in *Trim28*$^{Phd/+}$ *(PHD/+)* and in control (*Cont*) ovaries. Conversely, *Trim28*$^{Phd/cKO}$ (*PHD/cKO*) ovaries display disorganized follicles with appearance of pseudo-tubules, as observed in *Trim28*^{cKO} (*cKO*) ovaries. Scale bar 100µm. At least three independent biological replicates were analysed, and the images presented are representative of all replicates.

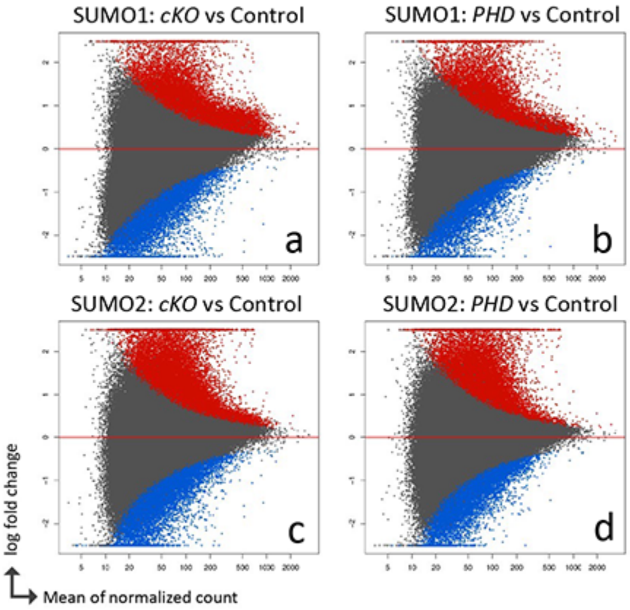

Fig. S16. MA plots for SUMO1 (**a** and **b**) and SUMO2 (**c** and **d**) showing the mean *versus* the ratio of normalized read counts. Spots corresponding to differential signal between mutant (*Trim28*^{cKO}*, cKO,* or *Trim28*$^{Phd/cKO}$, *PHD*) and control with Adj Pval<0.05 coloured in blue (hypo-SUMOylated) or red (hyper-SUMOylated).

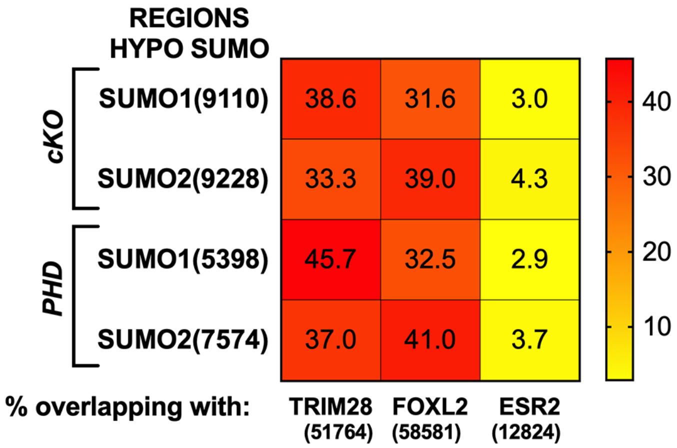

Supplementary Fig. 17. Percentage of hypo-SUMOylated ChIP-seq peaks (SUMO1 and SUMO2) in mutant ovaries (*Trim28*^{cKO}*, cKO,* and *Trim28*$^{Phd/cKO}$, *PHD*) that overlap with TRIM28 and FOXL2 peaks genome-wide in control ovaries. ESR2 peaks where obtained from Lindeman and colleagues[2] The values in brackets correspond to the number of peaks.

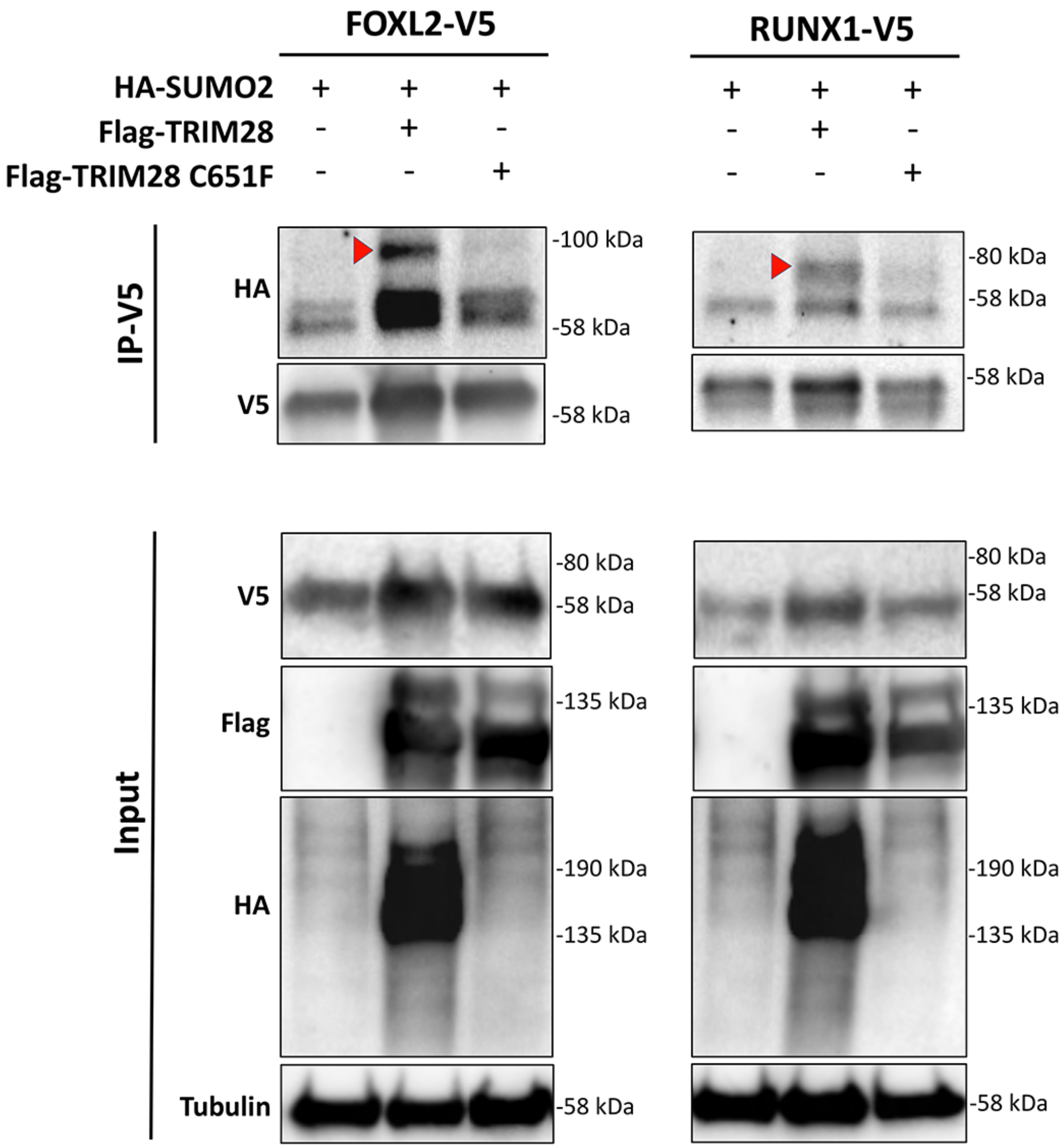

Supplementary Fig. 18: TRIM28 induces SUMOylation of FOXL2 and RUNX1. HEK293T cells were transfected with plasmids expressing V5-tagged (C-terminal) FOXL2 or RUNX1, HA-SUMO2, and Flag-tagged wild type TRIM28 or TRIM28 C651F. After 48h of transfection, cells were lysed in buffer with high concentration of SDS, diluted, and then immunoprecipitated with anti-V5 affinity resins. The IP complexes were analysed by western blotting with an anti-HA antibody to detect SUMOylated proteins and an anti-V5 antibody (upper panels: IP-V5). The IP complexes and cell lysates were also probed with the indicated antibodies to determine the protein input and the overall SUMOylation of cellular proteins (lower panels: Input). For both FOXL2 and RUNX1, the SUMOylated proteins of lower molecular weight correspond to the band detected by the V5 antibody suggesting that both proteins are SUMOylated by an endogenous E3-ligase. By contrast, in the presence of TRIM28, a SUMOylated form of higher molecular weight is detected for FOXL2 and RUNX1 (red triangle), but not in cells transfected with TRIM28 C651F. These results are representative of three independent experiments.

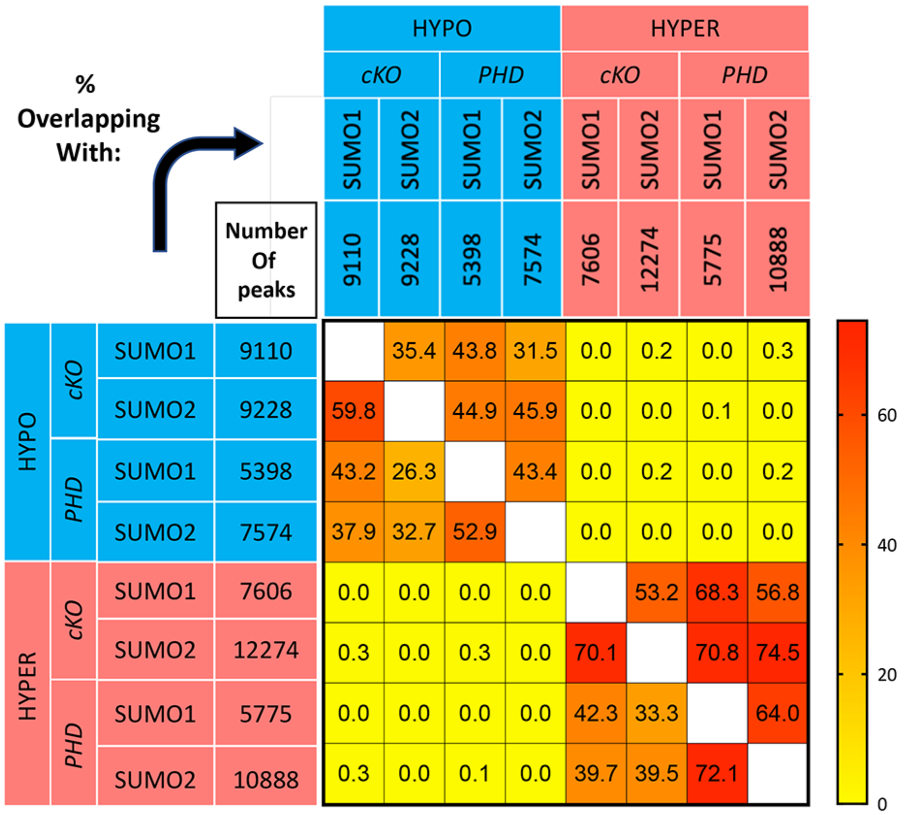

Supplementary Fig. 19. Percentage of overlap between the indicated peaks.

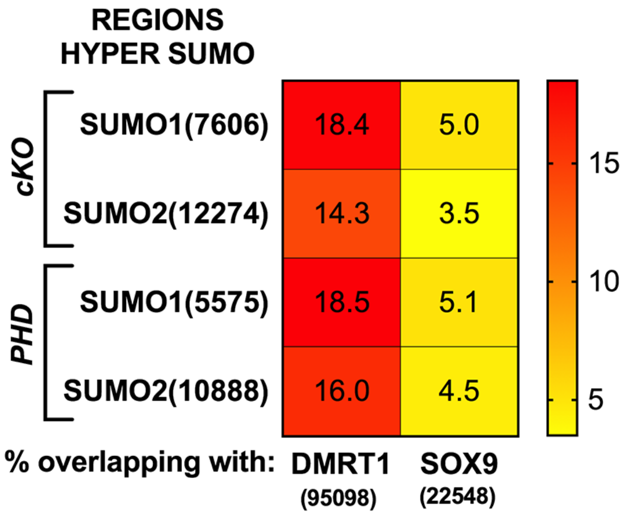

Supplementary Fig. 20. Percentage of hyper-SUMOylated ChIP-seq peaks (SUMO1 and SUMO2) in mutant ovaries (*Trim28^{cKO}*, *cKO,* and *Trim28$^{Phd/cKO}$*, *PHD*) that overlap with DMRT1and SOX9 peaks from sexual fate reprograming by the testicular transcription factor DMRT1 (*CAG-Stop-Dmrt1-Gfp; Nr5a1-Cre*) in adult ovary [2]. The values in brackets correspond to the number of peaks.

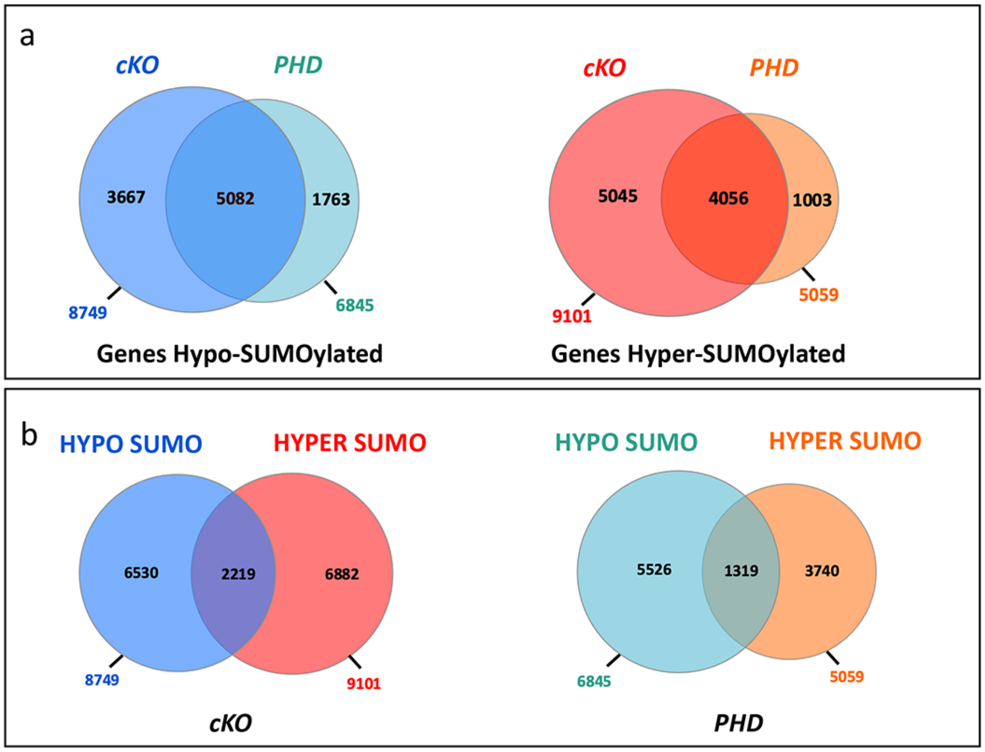

Supplementary Fig. 21. a, Venn diagrams comparing in *Trim28*^{cKO} and *Trim28*$^{Phd/cKO}$ ovaries (*cKO* and *PHD,* respectively) hypo-SUMOylated (left diagram) and hyper-SUMOylated (right diagram) genes. **b,** Venn diagrams comparing hypo- and/or hyper-SUMOylated genes in *Trim28*^{cKO} (*cKO*, left) and *Trim28*$^{Phd/cKO}$ (*PHD*; right) ovaries. In both mutants, some genes display both hypo- and hyper-SUMOylated peaks (2119 in cKO and 1319 in PHD). Data were obtained from Supplementary Data S7.

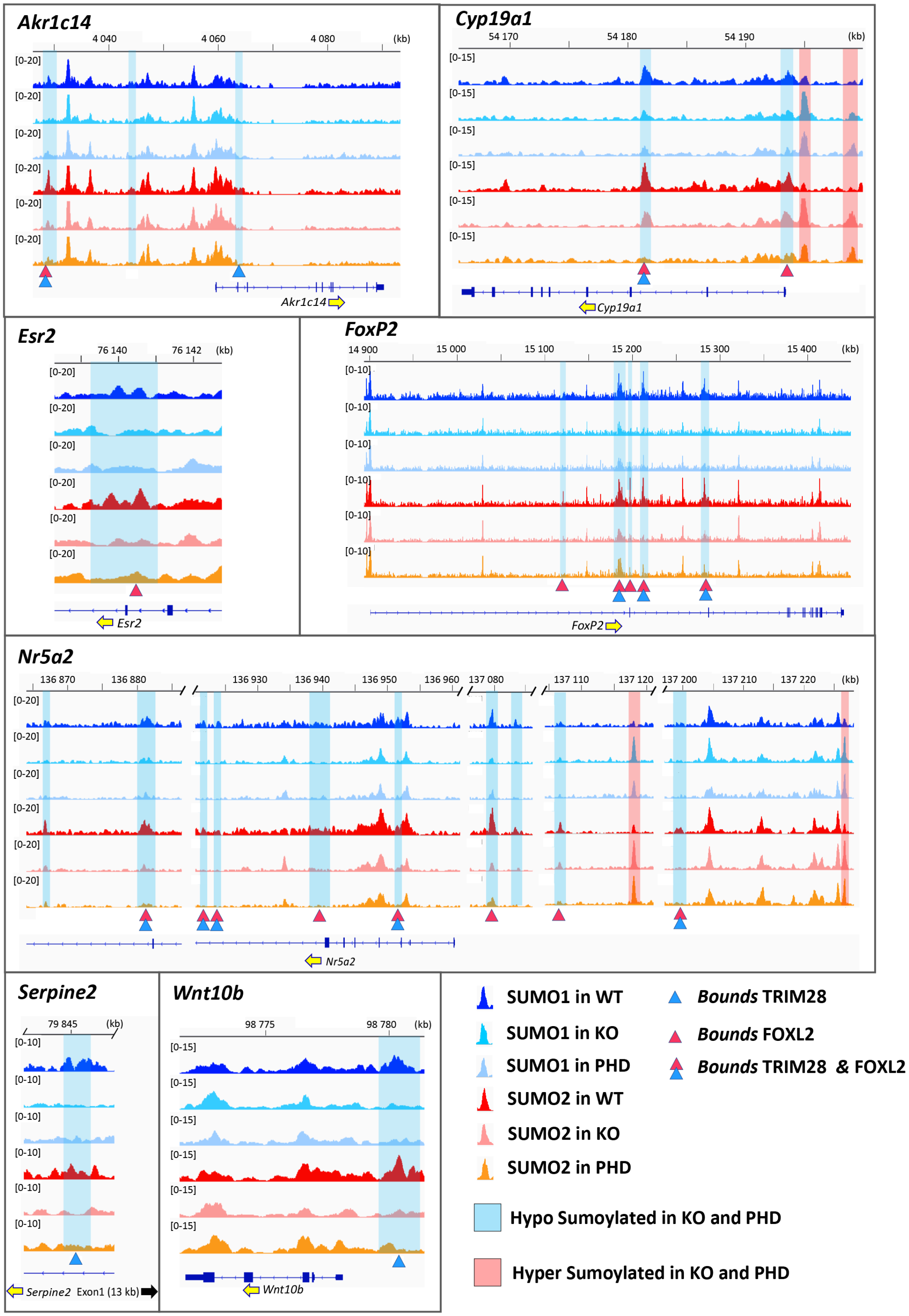

Supplementary Fig. 22. SUMOylation status (SUMO1 and 2) in *Trim28*^{cKO} and *Trim28*$^{Phd/cKO}$ ovaries (*cKO* and *PHD*, respectively) of genes that are downregulated in *Trim28*^{cKO} ovaries. Blue and red backgrounds highlight hypo-SUMOylated and hyper-SUMOylated regions, respectively. Blue and red triangles represent the centre of TRIM28 and FOXL2 peaks, respectively (see Supplementary Fig. 11).

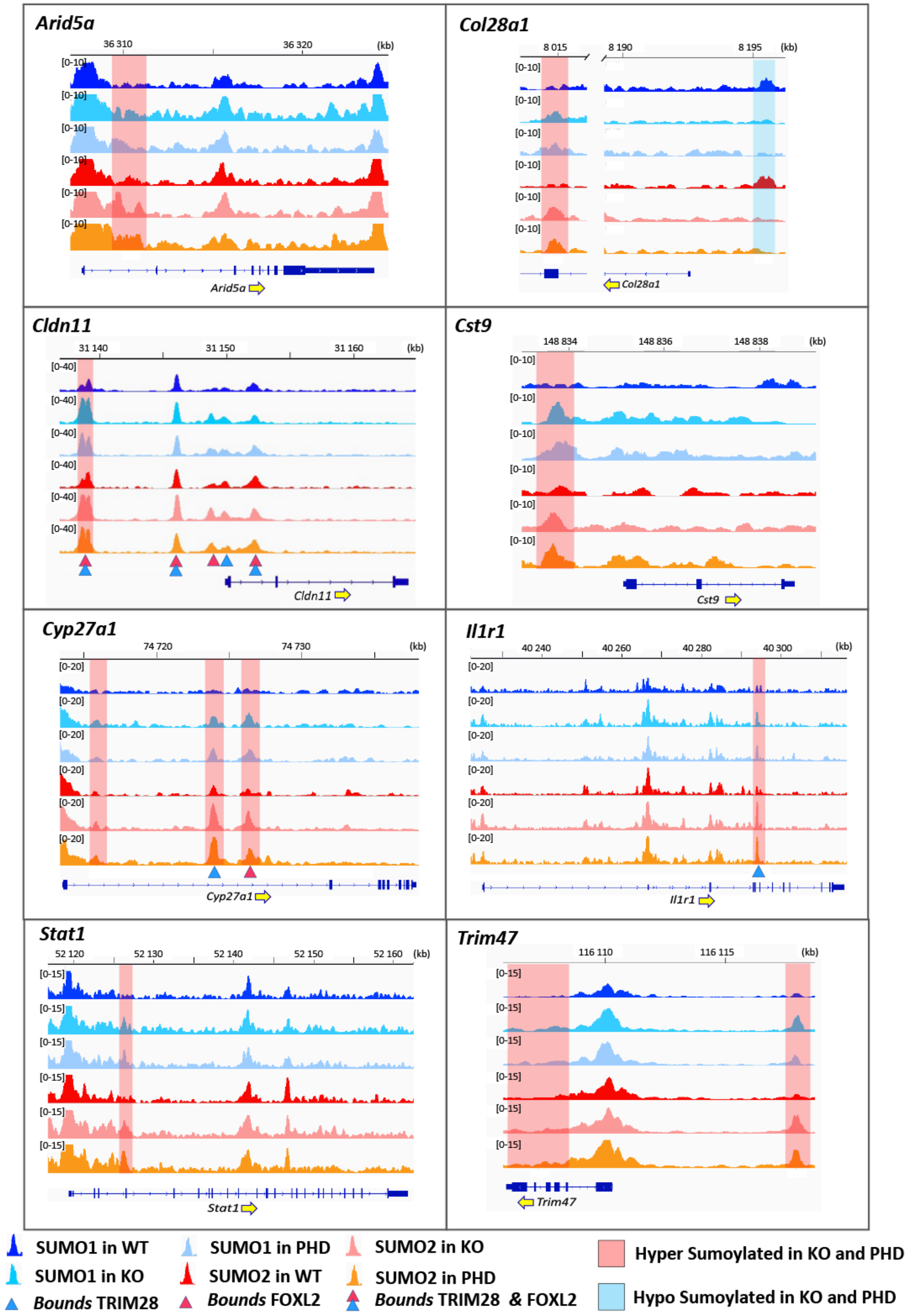

Supplementary Fig. 23. Same as in fig. S22, but for genes that are upregulated in *Trim28*^{cKO} ovaries. Blue and red backgrounds highlight hypo-SUMOylated and hyper-SUMOylated regions, respectively. Blue and red triangles represent the centre of TRIM28 and FOXL2 peaks, respectively (see fig S12).

Supplementary References

1. Love MI, Huber W, Anders S. Moderated estimation of fold change and dispersion for RNA-seq data with DESeq2. *Genome Biol* **15**, 550 (2014).

2. Lindeman RE*, et al.* The conserved sex regulator DMRT1 recruits SOX9 in sexual cell fate reprogramming. *Nucleic Acids Res* **49**, 6144-6164 (2021).
