# Supplementary material for: TRIM28-dependent SUMOylation protects the adult ovary from activation of the testicular pathway
Source: Nat Commun. 2022 Jul 29;13:4412. doi: 10.1038/s41467-022-32061-1 (PMC9338040; doi:10.1038/s41467-022-32061-1)
Supplement: Supplementary file 3 — Description of Additional Supplementary Files [file 41467_2022_32061_MOESM3_ESM.pdf]

Description of Additional Supplementary Files

Supplementary Data 1
Description: A large Excel Table containing three separate sheets.
-Genes down regulated in Trim28 cKO ovary (Log2 FC (control vs cKO)>1)
-Genes up regulated in Trim28 cKO ovary (Log2 FC (control vs cKO)<-1)
For each sheet, are available:
Log2 Fc
Adj P value
Gene bound by TRIM28 in control ovary (YES/NO)
Gene bound by FOXL2 in control ovary (YES/NO)

-Summary of the genes up and down regulated in cKO

Supplementary Data 2
Additional information on single cell RNAseq experiment.

Supplementary Data 3
An Excel table containing four separate sheets concerning scRNA seq analysis.
df_gene_clusters: description of the cell clusters with annotation and enriched gene names
upregulated_Granulosa
upregulated_Intermediate
upregulated_Sertoli

Supplementary Data 4
Enriched GO terms found from the intermediate cell population obtained from scRNA seq analysis

Supplementary Data 5
A large Excel Table containing two sheets for TRIM28 and FOXL2 ChIPseq made in control ovary.
Annotations TRIM28: Genomic Annotation using mm10 of TRIM28 ChIPseq peaks. Overlap with FOXL2 peaks. Neighbouring genes UP or Down regulated in TRIM28 cKO (Yes/No)
Annotations FOXL2: Genomic Annotation using mm10 of FOXL2 ChIPseq peaks. Overlap with TRIM28 peaks. Neighbouring genes UP or Down regulated in TRIM28 cKO (Yes/No)

Supplementary Data 6
A large Excel Table of differentially SUMOylated peaks (SUMO1 or SUMO2) with genomic annotation for cKO mutant ovary vs control, and PHD mutant ovary vs control.
Are available:
Hypo and Hyper-SUMOylation SUMO1 or SUMO2 in cKO and PHD mutant (YES/NO)
Log2 FC for each condition
Adjusted p-value
Neighbouring gene up or down in cKO (YES/NO)
Neighbouring gene bound TRIM28 (YES/NO)
Overlap of the SUMO peak with TRIM28 peak (bp)

Neighbouring gene bound FOXL2 (YES/NO)
Overlap of the SUMO peak with FOXL2 peak (bp)

Supplementary Data 7
Excel table gene lists from figure 3d and 5c
Sheet “Down in KO vs TRIM28 FOXL2” and Sheet “Up in KO vs TRIM28 FOXL2”: genes from figure 3d
Sheet “Down in KO vs SUMO” and sheet “Up in KO vs SUMO”: genes from figure 5c
