# Supplementary material for: TRIM28-dependent SUMOylation protects the adult ovary from activation of the testicular pathway
Source: Nat Commun. 2022 Jul 29;13:4412. doi: 10.1038/s41467-022-32061-1 (PMC9338040; doi:10.1038/s41467-022-32061-1)
Supplement: Supplementary file 11 — Reporting Summary [file 41467_2022_32061_MOESM11_ESM.pdf]

nature research

Corresponding author(s): Francis Poulat

Last updated by author(s): July 14 2022

Reporting Summary

Nature Research wishes to improve the reproducibility of the work that we publish. This form provides structure for consistency and transparency in reporting. For further information on Nature Research policies, see our Editorial Policies and the Editorial Policy Checklist.

Statistics

For all statistical analyses, confirm that the following items are present in the figure legend, table legend, main text, or Methods section.

n/a	Confirmed	
☐	☒	The exact sample size (*n*) for each experimental group/condition, given as a discrete number and unit of measurement
☐	☒	A statement on whether measurements were taken from distinct samples or whether the same sample was measured repeatedly
☐	☒	The statistical test(s) used AND whether they are one- or two-sided<br>*Only common tests should be described solely by name; describe more complex techniques in the Methods section.*
☒	☐	A description of all covariates tested
☐	☒	A description of any assumptions or corrections, such as tests of normality and adjustment for multiple comparisons
☐	☒	A full description of the statistical parameters including central tendency (e.g. means) or other basic estimates (e.g. regression coefficient) AND variation (e.g. standard deviation) or associated estimates of uncertainty (e.g. confidence intervals)
☐	☒	For null hypothesis testing, the test statistic (e.g. *F*, *t*, *r*) with confidence intervals, effect sizes, degrees of freedom and *P* value noted<br>*Give P values as exact values whenever suitable.*
☒	☐	For Bayesian analysis, information on the choice of priors and Markov chain Monte Carlo settings
☒	☐	For hierarchical and complex designs, identification of the appropriate level for tests and full reporting of outcomes
☒	☐	Estimates of effect sizes (e.g. Cohen's *d*, Pearson's *r*), indicating how they were calculated

Our web collection on statistics for biologists contains articles on many of the points above.

Software and code

Policy information about availability of computer code

Data collection	For sequencing, Image analysis and base calling were performed with RTA 2.7.3 and CASAVA 2.17.1.1.<br>Images were captured with a Zeiss LSM780 confocal microscope and analysed with the Imaris
Data analysis	For Chlpseq.<br>TRIM28 and FOXL2. Libraries were sequenced on SQ00/HS-1x50_40 — Hiseq 4000 sequencing 1x50 bases<br>SUMO1 and SUMO2. Libraries were sequenced on SQ00/HS-2x100_40 — Hiseq 4000 sequencing 2x100 bases<br>For TRIM28 and FOXL2 ChIP-seq: Reads were mapped to the Mus musculus genome (assembly mm10) using Bowtie43 v1.0.0 with default parameters except for "-p 3 -m 1 –strata –best –chunkmbs 128.<br>BAM files were sorted using SAMtools44 v0.1.19. The tool rmDupByMids.pl provided by Active Motif was then used to remove duplicated reads. Peaks were called using MACS2 with default parameters except for "-g mm -f BAM –broad –broad-cuto 0.1 –keep-dup all".<br>For SUMO1 and SUMO2 ChIP-seq: Reads were mapped to the Mus musculus genome (assembly mm10) using Bowtie v1.0.0 with default parameters except for "-p 3 -m 1 –strata –best".<br>BAM files were sorted using SAMtools v0.1.19. The tool rmDupByMids.pl provided by Active Motif was used to remove duplicated reads.<br>Peak calling data were analyzed using the Encode ChIP-seq pipeline v1.3.6. Peak caller: spp v1.15.5.<br>Peaks were annotated relative to genomic features using Homer v4.11.0 with Ensembl v92 annotations.<br>For all proteins of interest, all detected peaks were combined to merge all peaks (249760 peaks) using Bedtools merge v2.26.0.<br>Comparisons were performed using the method implemented in the DESeq2 Bioconductor library (DESeq2 v1.6.3).<br>Heatmap analyses were performed with SeqMINER v1.3.3g.<br>For Single cell RNAseq<br>Single-cell sequencing was performed on the 10X Genomic platform.<br>After converting the base calls to FASTQ format, reads were processed with the CellRanger v3 count module. This aligned reads with STAR to the GRCm38 reference genome using the M15 (Ensembl 90) GENCODE annotation, and then derived the gene vs. cell expression counts

matrices. These pre-processing steps were performed on the Baobab HPC cluster at the University of Geneva
Using Scanpy with Anndata, matrices were concatenated across all samples, and cells with <50 genes expressed were removed (and genes expressed in <3 cells). The top 50 PCA components were embedded in the neighborhood graph with batch correction via BBKNN58 with the neighbors_within_batch=5 parameter that removed batch effects among biological replicates . Data were visualized in the transcriptional space with 2D uniform manifold approximation and projection (UMAP) and force directed graphs (FDG) using the ForceAtlas2 implementation60, mainly in Jupyter notebooks
RNaseq:
Libraries were sequenced on an Illumina HiSeq 4000 system using single-end 1 × 50 bp. Image analysis and base calling were performed with RTA 2.7.3 and CASAVA 2.17.1.1.Reads were mapped to the mm10 assembly of the Mus musculus genome using STAR version 2.5.3a. Gene expression quantification was performed from uniquely aligned reads using htseq-count version 0.6.1p1, with annotations from Ensembl version 92 and "union" mode. Comparison between wild type and cKO samples was performed using the Wald test for differential expression proposed by Love et al.48 and implemented in the Bioconductor package DESeq2 version 1.16.1.
Quantification of fluorescence for SUMO1/2 staining: Imaris V9.5

For manuscripts utilizing custom algorithms or software that are central to the research but not yet described in published literature, software must be made available to editors and reviewers. We strongly encourage code deposition in a community repository (e.g. GitHub). See the Nature Research guidelines for submitting code & software for further information.

Data

Policy information about availability of data

All manuscripts must include a data availability statement. This statement should provide the following information, where applicable:
- Accession codes, unique identifiers, or web links for publicly available datasets
- A list of figures that have associated raw data
- A description of any restrictions on data availability

All data are available in the main text or supplementary materials. RNA-seq and ChIP-seq data have been deposited in the Gene Expression Omnibus under accession number GSE166385 (RNA-seq and ChIP-seq) and GSE166444 (scRNA-seq) and the mass spectrometry proteomic data have been deposited in the ProteomeXchange Consortium via the PRIDE partner repository with the dataset identifier PXD024439. Raw data underlying all reported mean values in graphs are provided in the Source Data File. All other relevant data supporting the key findings of this study are available in the supplementary files.

Field-specific reporting

Please select the one below that is the best fit for your research. If you are not sure, read the appropriate sections before making your selection.

☒ Life sciences ☐ Behavioural & social sciences ☐ Ecological, evolutionary & environmental sciences

For a reference copy of the document with all sections, see nature.com/documents/nr-reporting-summary-flat.pdf

Life sciences study design

All studies must disclose on these points even when the disclosure is negative.

Sample size	All experiments using mouse samples were performed on at least 3 biological replicates.
Data exclusions	No data were excluded from the analyses.
Replication	Immunofluorescence / histological experiments were replicated at least 3 times using independent biological replicates and attempts at replication of the results were successful. RT-qPCR were preformed independently from at least 3 animals (gonad pairs). For quantification of steroids from gonads, 3-4 pairs of gonad were analysed independently. For SUMOylation assay in HEK293T cells, 3 independent experiments were performed giving the same results.
Randomization	samples were allocated into experimental group by genotype.
Blinding	For the biological experiments, investigators were not blinded to group allocation for data collection and analysis since the same investigator designed and performed the experiments.

Reporting for specific materials, systems and methods

We require information from authors about some types of materials, experimental systems and methods used in many studies. Here, indicate whether each material, system or method listed is relevant to your study. If you are not sure if a list item applies to your research, read the appropriate section before selecting a response.

Materials & experimental systems

n/a	Involved in the study
☐	☒ Antibodies
☐	☒ Eukaryotic cell lines
☒	☐ Palaeontology and archaeology
☐	☒ Animals and other organisms
☒	☐ Human research participants
☒	☐ Clinical data
☒	☐ Dual use research of concern

Methods

n/a	Involved in the study
☐	☒ ChIP-seq
☐	☐ Flow cytometry
☒	☐ MRI-based neuroimaging

Antibodies

Antibodies used

FOXL2 (rabbit) Immunofluorescence: 1/400. ChIpseq: 2µg/IP. Provided by Dagmar Wilhem (Co-author) ref DOI: 10.1016/j.mod.2009.02.006
SOX9 (rabbit) . Immunofluorescence : 1/400. Producted in our laboratory. ref: DOI: 10.1073/pnas.172383099
SOX8 (guinea pig) . Immunofluorescence : 1/300. Provided by Michael Wegner. Ref DOI: 10.1016/j.ydbio.2005.03.010
DMRT1 (rabbit) . Immunofluorescence: 1/400. Provided by David Zarkower. Ref DOI: 10.1016/j.ydbio.2013.02.014
TRIM28 (mouse) . Immunofluorescence: 1/1000. Provided by Florence Cammas (Co-author). Ref DOI: 10.1242/jcs.115.17.3439
TRIM28 (rabbit). ChIpseq 2µg/IP. Provided by Florence Cammas (co-author). Ref DOI: 10.1091/mbc.e08-05-0510
SUMO-1 and SUMO-2/3: immunofluorescence: 1/300. Provided by Guillaume Bossis (co-author)
SUMO-2/3: immunofluorescence: 1/300 . Provided by Guillaume Bossis (co-author)
V5-HRP: Invitrogen. Ref P/F 46-0708. Western blotting:1/5000
HA-HRP:. Invitrogen. Ref 26183-HRP. Western blotting: 1/5000
V5: Invitrogen. Ref 37-7500. Western blotting: 1/2000.
HA: Invitrogen. Ref MA1-12429. Western blotting: 1/2000.
FLAG: Invitrogen. Ref MA1-91878. Western blotting: 1/3000
Tubulin: Sigma. Ref T9026. Western blotting: 1/3000

Validation

Antibody validation is based on previously published data.
FOXL2 (rabbit) (Wilhelm, D. et al. 2009. DOI: 10.1016/j.mod.2009.02.006)
SOX9 (rabbit) (Gasca, S. et al 2002, DOI: 10.1073/pnas.172383099)
SOX8 (guinea pig) (Stolt, C.C et al 2005, DOI: 10.1016/j.ydbio.2005.03.010)
DMRT1 (rabbit) (Krentz, A.D. et al 2013, DOI:10.1016/j.ydbio.2013.02.014)
TRIM28 (mouse) (Cammas, F. et al 2002, DOI: 10.1242/jcs.115.17.3439).
TRIM28 (rabbit) (Riclet, R. et al. 2009 . DOI: 10.1091/mbc.E08-05-0510).
Polyclonal antibodies against SUMO-1 and SUMO-2/3 were produced by injecting rabbits with recombinant His-tagged mouse SUMO-1 and SUMO-3 proteins produced in bacteria and purified using Ni-NTA column followed by Superdex 75 gel filtration, as previously described (see methods). Rabbit sera were affinity-purified using GST-SUMO1 or GST-SUMO2 coupled to CnBr-activated Sepharose (SIGMA). Their specificity was confirmed by immunoblotting using recombinant mouse SUMO-1 and SUMO-2.
The other antibodies were purchased from commercial sources, and validated by the manufacturers.
Anti-V5 HRP: https://www.thermofisher.com/antibody/product/V5-Tag-Antibody-Monoclonal/R961-25
Anti-Ha HRP: https://www.thermofisher.com/antibody/product/HA-Tag-Antibody-clone-2-2-2-14-Monoclonal/26183-HRP
Anti-V5: https://www.thermofisher.com/antibody/product/V5-Tag-Antibody-clone-2F11F7-Monoclonal/37-7500
Anti-Ha: https://www.thermofisher.com/antibody/product/HA-Tag-Antibody-clone-12CA5-Monoclonal/MA1-12429
Anti-Flag: https://www.thermofisher.com/antibody/product/DYKDDDDK-Tag-Antibody-clone-FG4R-Monoclonal/MA1-91878
Anti-tubulin: https://www.sigmaaldrich.com/FR/fr/product/sigma/t9026

Eukaryotic cell lines

Policy information about cell lines

Cell line source(s)	HEK 293T were obtained from American tissue culture collection (ATCC) (ref CRL-1573)
Authentication	None
Mycoplasma contamination	Cells were routinely tested negative for micoplasma contamination
Commonly misidentified lines (See ICLAC register)	Nothing found

Animals and other organisms

Policy information about studies involving animals; ARRIVE guidelines recommended for reporting animal research

Laboratory animals	Sf1-CreTg/Tg mice (B6D2-Tg(Nr5a1-cre)2Klp) were provided by late Dr. Keith Parker. The Trim28 conditional allele was provided by Florence Cammas. To generate granulosa Trim28 conditional knock-out mice, mice carrying Trim28 loxP-flanked alleles (flox) (Trim28flox/flox) were crossed with mice bearing the Nr5a1:Cre transgene to generate Trim28flox/+; Nr5a1:Cre mice. These mice were then crossed with Trim28flox/flox mice to generate Trim28flox/flox; Nr5a1:Cre female mice; these mice were referred to as Trim28cKO null mutants. These crosses also generated Trim28flox/flox mice without the Nr5a1:Cre transgene and Trim28flox/+; Nr5a1:Cre mice; both genotypes did not show any histological defect and were thus referred to as control mice.<br>The Trim28Phd/+ mutant mouse line was established at the MCI/ICS (Mouse Clinical Institute - Institut Clinique de la Souris-, Illkirch, France; http://www-mci.u-strasbg.fr). Trim28Phd/+ embryonic stem cells were used to derive Trim28Phd/+ mice. To generate granulosa knock-in mice that express only the mutant TRIM28 protein, Trim28Phd/+ mice and mice bearing the Nr5a1:Cre transgene were first intercrossed to produce Trim28Phd/+: Nr5a1:Cre mice. These mice were then crossed with Trim28flox/flox mice to generate Trim28Phd/flox: Nr5a1:Cre mice; these mice were referred to as Trim28Phd/cKo knock-in mutants or PHD mutants. These crosses also generated TrimPhd/flox mice without the Nr5a1:Cre transgene, Trim28Phd/+; Nr5a1:Cre mice, and Trim28flox/+; Nr5a1:Cre mice that did not have any histological defect, and were referred to as control mice.<br>Mice of both sexes were used. Age 4 dpp, 20 dpp, 2, 4 and 7 month.Embryos of E13.5 and E18.8 were also used<br>All the mice were housed with a 12h light-dark cycle, and had a temperature of 22-24°C with 50–60% humidity
Wild animals	The study did not involve wild animals.
Field-collected samples	The study did not involve samples collectd from the field.
Ethics oversight	The preliminary experiments showed that the phenotypes observed were not harmful for the animals, as no experiment was carried out on living animals, ethical approval was not required. Animals where only bred, and tissues collected after euthanasia following recommendation to the “Reseau des Animaleries de Montpellier” (RAM) guidelines. Animal care and handling were according to the RAM guidelines. Experiments were designed following the European regulations for the care and use of animals in order to protect vertebrates animals for experimental and other scientific purpose (Directive 89/609)

Note that full information on the approval of the study protocol must also be provided in the manuscript.

ChIP-seq

Data deposition

- [x] Confirm that both raw and final processed data have been deposited in a public database such as GEO.
- [x] Confirm that you have deposited or provided access to graph files (e.g. BED files) for the called peaks.

Data access links<br>*May remain private before publication.*	GSE166385; https://www.ncbi.nlm.nih.gov/geo/
Files in database submission	ChIPseq_TRIM28: GSM5070257_wigs_for_FSPT1.wig.gz 241.9 Mb WIG<br>ChIPseq_FOXL2: GSM5070258_wigs_for_FSPT2.wig.gz 251.2 Mb WIG<br>Input ChIPseq TRIM28 and FOXL2: GSM5070259_wigs_for_FSPT12.wig.gz 234.2 Mb WIG<br>Chipseq_WT_SUMO1_1: GSM5070260_wigs_for_FSPT21.wig.gz 234.3 Mb) WIG<br>Chipseq_WT_SUMO1_2: GSM5070261_wigs_for_FSPT22.wig.gz 243.2 Mb WIG<br>Chipseq_WT_SUMO2_1: GSM5070262_wigs_for_FSPT23.wig.gz 219.3 Mb WIG<br>Chipseq_WT_SUMO2_2: GSM5070263_wigs_for_FSPT24.wig.gz 231.8 Mb WIG<br>Input ChIPseq SUMO1 and 2 WT: GSM5070272_wigs_for_FSPT34.wig.gz 247.1 Mb WIG<br>Chipseq_KO_SUMO1_1: GSM5070264_wigs_for_FSPT25.wig.gz 228.1 Mb WIG<br>Chipseq_KO_SUMO1_2: GSM5070265_wigs_for_FSPT26.wig.gz 236.1 Mb WIG<br>Chipseq_KO_SUMO2_1: GSM5070266_wigs_for_FSPT27.wig.gz 222.1 Mb WIG<br>Chipseq_KO_SUMO2_2: GSM5070267_wigs_for_FSPT28.wig.gz 218.7 Mb WIG<br>Input ChIPseq SUMO1 and 2 KO: GSM5070273_wigs_for_FSPT35.wig.gz 249.4 Mb WIG<br>Chipseq_PHD_SUMO1_1: GSM5070268_wigs_for_FSPT29.wig.gz 242.0 Mb WIG<br>Chipseq_PHD_SUMO1_2: GSM5070269_wigs_for_FSPT30.wig.gz 230.3 Mb WIG<br>Chipseq_PHD_SUMO2_1: GSM5070270_wigs_for_FSPT31.wig.gz 206.9 Mb WIG<br>Chipseq_PHD_SUMO2_2: GSM5070271_wigs_for_FSPT32.wig.gz 226.4 Mb WIG<br>Input ChIPseq SUMO1 and 2 PHD: GSM5070274_wigs_for_FSPT36.wig.gz 242.4 Mb WIG
Genome browser session<br>(e.g. UCSC)	https://genome.ucsc.edu/s/Slegras/PoulatNature

Methodology

Replicates	For TRIM28 and FOXL2 ChIP-seq: Each ChIP-seq library was prepared from a pool of three independent IPs, and each IP was prepared with ovaries of two different animals.<br>For SUMO1 and SUMO2 ChIP-seq: Two independent ChIP-seq libraries were prepared. For each library three independent IPs were pooled, each IP prepared from ovaries of two different animals.
Sequencing depth	For TRIM28 and FOXL2 Chipseq and corresponding input:<br>ChIP-seq libraries were sequenced as single-end 1x50 bases on Illumina<br>Hiseq 4000<br>TRIM28. Total number of reads: 81107892. Uniquely mapped non-duplicate: 59573052<br>FOXL2. Total number of reads: 94573010. Uniquely mapped non-duplicate: 72037849<br>Input. Total number of reads: 59284485. Uniquely mapped non-duplicate: 41577921<br><br>FOR SUMO1 and SUMO2 ChIPseq in WT, cKO, PHD mutants (in duplicates) and and corresponding input:<br>ChIP-seq libraries were sequenced as single-end 2x100 bases on Illumina<br>Hiseq 4000<br>WT_SUMO1_1. Total number of reads: 42905465. Uniquely mapped non-duplicate: 32400051<br>WT_SUMO1_2. Total number of reads: 47160196. Uniquely mapped non-duplicate: 35657386<br>WT_SUMO2_1. Total number of reads: 42621289. Uniquely mapped non-duplicate: 33255043<br>WT_SUMO2_2. Total number of reads: 47465742. Uniquely mapped non-duplicate: 36657385<br>KO_SUMO1_1. Total number of reads: 43225759. Uniquely mapped non-duplicate: 31377715<br>KO_SUMO1_2. Total number of reads: 47447331. Uniquely mapped non-duplicate: 36232718<br>KO_SUMO2_1. Total number of reads: 48793879. Uniquely mapped non-duplicate: 38678607<br>KO_SUMO2_2. Total number of reads: 44994645. Uniquely mapped non-duplicate: 35321197<br>PHD_SUMO1_1. Total number of reads: 50983110. Uniquely mapped non-duplicate: 37387183<br>PHD_SUMO1_2. Total number of reads: 41760456. Uniquely mapped non-duplicate: 31033559<br>PHD_SUMO2_1. Total number of reads: 38905172. Uniquely mapped non-duplicate: 30058010<br>PHD_SUMO2_2. Total number of reads: 54628693. Uniquely mapped non-duplicate: 42141622<br>INPUT_WT. Total number of reads: 43623160. Uniquely mapped non-duplicate: 33679794<br>INPUT_WT. Total number of reads: 48033987. Uniquely mapped non-duplicate: 37356927<br>INPUT_WT. Total number of reads: 40780979. Uniquely mapped non-duplicate: 31556862
Antibodies	see before for references.<br>FOXL2 (rabbit) ChIP-seq and ChIP-SICAB: 5µl (2µg) / IP.<br>TRIM28 (rabbit) . ChIP-seq: 2µg / IP.<br>SUMO-1. ChIPseq: 2µg /IP<br>SUMO-2/3. ChIPseq: 2µg /IP
Peak calling parameters	For TRIM28 and FOXL2 ChIP-seq, Peaks were called using MACS2 with default parameters except for "-g mm -f BAM –broad –broad-cuto 0.1 –keep-dup all". For SUMO1 and SUMO2 ChIP-seq: For peak calling data were analyzed using the Encode ChIP-seq pipeline v1.3.6. Peak caller: spp v1.15.5 (Kharchenko, P. V., Tolstorukov, M. Y. & Park, P. J. Design and analysis of ChIP-seq experiments for DNA-binding proteins. Nat Biotechnol 26, 1351-1359, doi:10.1038/nbt.1508 (2008).) Spp v1.15.5 was run through the ENCODE pipeline v1.3.6 using the following parameters "-npeak=300000 -fdr=0.01".
Data quality	ChIP-seq libraries were filtered to retain only reads with average base quality score >20. Reads were mapped to the Mus musculus genome (assembly mm10) using Bowtie v1.0.0. The tool rmDupByMids.pl provided by Active Motif was then used to remove duplicated reads.
Software	Bowtie v1.0.0.<br>SAMtools v0.1.19.<br>rmDupByMids.pl<br>MACS2.<br>Encode ChIP-seq pipeline v1.3.6. Peak caller: spp v1.15.545.<br>Homer v4.11.0<br>Bedtools merge v2.26.0.<br>DESeq2 v1.6.3<br>SeqMINER v1.3.3g.<br>RTA 2.7.3<br>CASAVA 2.17.1.1<br>STAR version 2.5.3a<br>DESeq2 version 1.16.1.<br>Bedtools intersect v2.26.0<br>R v4.1.1

Flow Cytometry

Plots

Confirm that:

☐ The axis labels state the marker and fluorochrome used (e.g. CD4-FITC).

☐ The axis scales are clearly visible. Include numbers along axes only for bottom left plot of group (a 'group' is an analysis of identical markers).

☐ All plots are contour plots with outliers or pseudocolor plots.

☐ A numerical value for number of cells or percentage (with statistics) is provided.

Methodology

Sample preparation	*Describe the sample preparation, detailing the biological source of the cells and any tissue processing steps used.*
Instrument	*Identify the instrument used for data collection, specifying make and model number.*
Software	*Describe the software used to collect and analyze the flow cytometry data. For custom code that has been deposited into a community repository, provide accession details.*
Cell population abundance	*Describe the abundance of the relevant cell populations within post-sort fractions, providing details on the purity of the samples and how it was determined.*
Gating strategy	*Describe the gating strategy used for all relevant experiments, specifying the preliminary FSC/SSC gates of the starting cell population, indicating where boundaries between "positive" and "negative" staining cell populations are defined.*

☐ Tick this box to confirm that a figure exemplifying the gating strategy is provided in the Supplementary Information.
